# The PGE_2_/IL-10 Axis Determines Susceptibility of B-1 Cell-Derived Phagocytes (B-1CDP) to *Leishmania major* Infection

**DOI:** 10.1371/journal.pone.0124888

**Published:** 2015-05-01

**Authors:** Angélica F. Arcanjo, Isabel F. LaRocque-de-Freitas, Juliana Dutra B. Rocha, Daniel Zamith, Ana Caroline Costa-da-Silva, Marise Pinheiro Nunes, Fabio P. Mesquita-Santos, Alexandre Morrot, Alessandra A. Filardy, Mario Mariano, Christianne Bandeira-Melo, George A. DosReis, Debora Decote-Ricardo, Célio Geraldo Freire-de-Lima

**Affiliations:** 1 Instituto de Biofísica Carlos Chagas Filho, Universidade Federal do Rio de Janeiro, Rio de Janeiro, Brazil; 2 Departamento de Veterinária, Universidade Federal Rural do Rio de Janeiro, Rio de Janeiro, Brazil; 3 Instituto Oswaldo Cruz, FIOCRUZ, Rio de Janeiro, Brazil; 4 Instituto de Microbiologia Paulo de Góes, Universidade Federal do Rio de Janeiro, Rio de Janeiro, Brazil; 5 Departmento de Microbiologia, Imunologia e Parasitologia, Universidade Federal de São Paulo, São Paulo, Brazil; Federal University of São Paulo, BRAZIL

## Abstract

B-1 cells can be differentiated from B-2 cells because they are predominantly located in the peritoneal and pleural cavities and have distinct phenotypic patterns and activation properties. A mononuclear phagocyte derived from B-1 cells (B-1CDP) has been described. As the B-1CDP cells migrate to inflammatory/infectious sites and exhibit phagocytic capacity, the microbicidal ability of these cells was investigated using the *Leishmania major* infection model *in vitro*. The data obtained in this study demonstrate that B-1CDP cells are more susceptible to infection than peritoneal macrophages, since B-1CDP cells have a higher number of intracellular amastigotes forms and consequently release a larger number of promastigotes. Exacerbated infection by *L*. *major* required lipid bodies/PGE_2_ and IL-10 by B-1CDP cells. Both infection and the production of IL-10 were decreased when PGE_2_ production was blocked by NSAIDs. The involvement of IL-10 in this mechanism was confirmed, since B-1CDP cells from IL-10 KO mice are more competent to control *L*. *major* infection than cells from wild type mice. These findings further characterize the B-1CDP cells as an important mononuclear phagocyte that plays a previously unrecognized role in host responses to *L*. *major* infection, most likely via PGE_2_-driven production of IL-10.

## Introduction

Macrophages represent distinct cells with phagocytic activity distributed through all tissues. Originally tissue-resident macrophages were assumed to be derived from circulating monocytes [1] differentiated from bone marrow progenitors. Recently, a change in this dogma was provided with conclusive evidences for the existence of a monocyte-independent differentiation pathway of resident macrophages, leading to shift in the paradigm of this model [2,3].

Recently, other studies have suggested that other cell lines could originate phagocytic macrophages [4,5]. These studies are based on previous experiments that demonstrated that B-1 cells present in mice and humans could differentiate into cells with characteristics similar to macrophages. Borrello and Phipps demonstrated that B-1 cells from the peritoneal cavity of mice differentiate into a phagocytic cell similar to macrophage-like cells [6]. Differentiation decreases immunoglobulin M expression but the expression of rearranged VH11 or VH12, heavy chain genes persist [7]. Graf et al demonstrated that B/macrophage cells express COX-1, and up-regulate COX-2 expression and prostaglandin E_2_ production in response to pro-inflammatory signals [8].

Several studies investigated the origin [9–12], immunological properties [9,13–18] and the involvement these cells in inflammatory reactions [15,19–28]. Despite the great interest on this cell type, little is known about B-1 cells and mainly on B-1 cell derived phagocytes (B-1CDP) in models of infections by microorganisms [7,21,29,30].

*L*. *major* is a protozoan parasite transmitted by sandflies of the genus *Lutzomyia* that inject the promastigote form into the dermis of the host. Once injected, the parasite is rapidly enclosed by phagocytic cells and transforms into the replicative intracellular amastigote form [31]. In susceptible hosts, such as BALB/c mice, *L*. *major* elicits a Th2 immune response and induces a progressive infection. In susceptible hosts, macrophages produce anti-inflammatory factors, such IL-10, TGF-β and PGE_2_, which act in favor of the protozoan [32].

Based on these data, we decided to investigate the interaction of B-1CDP cells from BALB/c mice with *L*. *major* to elucidate the possible influence of these cells on the progression of infection *in vitro*.

## Material and Methods

### Ethics statement

This study was carried out in strict accordance with the recommendations in the Guide for the Care and Use of Laboratory Animals of the National Institutes of Health (USA). The protocol was approved by the Committee on the Ethics of Animal Experiments of the Health Science Center of the Federal University of Rio de Janeiro (CEUA-CCS, Permit Number: IMPPG 038-05/16) and all efforts were made to minimize suffering.

### Mice and parasite

BALB/c mice of both sexes, aging 6–8 wk, were from Oswaldo Cruz Institute Animal Care Facility (Fiocruz, Rio de Janeiro, Brazil) C57BL/6 IL-10 Knock-out (KO) and C57BL/6 wild-type mice were kindly donated by Professor João Santana Silva from the Department of Pharmacology, School of Medicine, USP, Ribeirão Preto. *L*. *major* strain LV39 (MRHO/Sv/59/P) was isolated monthly from footpads of infected BALB/c mice and maintained *in vitro* as proliferating promastigotes. Parasites were maintained in Schneider medium (Life Technologies) supplemented with 10% FCS, 1% glutamine and 2% human urine.

### Cell culture

B-1CDP cells obtained as previously described [33]. Briefly, resident peritoneal cells were collected from peritoneal washouts of BALB/c mice. Cells (2 X 10^6^) were dispensed on 10 cm diameter plastic plates and the cultures incubated ay 37°C in 7% CO_2_ for 1h. After incubation, the culture supernatants were aspirated to remove non-adherent cells. Adherent monolayers were rinsed with antibiotic-free RPMI-1640 medium (Sigma), contained 15 mM HEPES, 2g of sodium bicarbonate/liter, 1mM L-glutamine and kept in 0,5 ml of antibiotic-free RPMI medium plus 10% fetal bovine serum for 6 days. B1 cells present in the supernatant of these cultures were aspirated, centrifuged, re-suspended in RPMI medium plus 1 0% fetal bovine serum and dispensed on cover slips in the bottom of 6 well plates. After 3 days in culture B-1CDP, adherent to the glass surface were removed from the substrate by ice-cold phosphate-buffered saline. Cells were counted, added (2 X 10^5^) to glass cover slips inserted in 24-well tissue culture plates. Peritoneal macrophages cultures were made as above described using adherent cells from the peritoneal cavity of BALB/c. Peritoneal macrophages were counted, added (2 X 10^5^) to glass cover slips inserted in 24-well tissue culture plates.

### Infection

B-1CDP cells and peritoneal macrophages were plated in 24 wells tissue culture plates (Nunc, Roskilde, Denmark) at 2 X 10^5^ cells/well in RPMI medium plus 10% fetal bovine serum. Cells immediately received 1X10^6^ stationary phase *L*. *major* promastigote, and were incubated in medium 10% FCS at 37°C. After 4 hours, monolayers were extensively washed with warm HBSS, to remove extracellular parasites. All cultures were done in medium 1% Nutridoma-SP, instead of FCS.

### Antibodies, cytokines and inhibitors

B-1CDP cells or peritoneal macrophage monolayers were treated with 1 μg/mL indomethacin, 10 μg/mL Aspirin (Sigma-Aldrich) or 1 μM/mL NS-398 (CaymanChem), or equivalent dosage of solvent (DMSO). Neutralizing anti-TGF-β and normal chicken IgY (R&D System), anti-IL-10 and rat IgG1 isotype control (BioSource Europe, Nivelles), were used at 10 μg/mL.

### Assessment of intracellular load of *L*. *major*

After 3 days, infected B-1CDP cells and macrophages monolayers were extensively washed, and medium was replaced by 0,5 ml of Schneider medium (Life Technologies), supplemented with 20% FCS and 2% human urine [34]. Monolayers were cultured at 26°C for additional 3 days. Intracellular load of *L*. *major* amastigote was estimated by production of proliferating extracellular motile promastigote in Schneider medium [34]. Alternatively, infected B-1CDP cells and peritoneal macrophages were cultured on glass coveslips place inside culture vessels (Corning) at 37°C in 7% CO_2_. After 4 days, coverslips were washed and stained with May-Grunwald Giemsa (Sigma-Aldrich), and intracellular amastigotes were counted in 100 B-1CDP cells or macrophages. Results are shown as amastigote number per phagocyte, and as percentage of infected macrophages. All results are mean and SE of triplicated cultures.

### Lactate Dehydrogenase Assay

In order to evaluate the integrity infected B-1CDP cells and peritoneal macrophages, an aliquot of the supernatant was withdrawn and frozen for determination of LDH release. LDH activity was evaluated by using an assay kit (Doles Reagents, Brazil), which measure the amount of a colored complex derived from the NADH formed by the enzymatic reaction, using a spectrophotometric method (*A*_490_).

### Immunofluorescence Microscopy

Infected B-1CDP cells and macrophages were obtained as described above. After different time in culture, cells were fixed for 1 h in 3.5% formalin. After fixation, cells were washed in PBS containing 1% BSA (Sigma) to block free sites and permeabilized by treatment with 0.05% saponin (Sigma) for 30 min. Polyclonal goat antibody anti-mouse IgM-FITC (μ chain specific) (Sigma) (1:1000) and biotin rat antibody anti-mouse F4/80 was diluted in PBS/BSA/saponin and incubated for 1 h at room temperature. After three washes in the same solution, glass cover slips were incubated with anti-rat IgG-FITC (F4/80). After incubation, glass cover slips were washed again and incubated in 1 μg /mL of DAPI. After 10 min of incubation, glass cover slips were washed, mounted in 0.1 M glycerol and sealed with nail varnish. Images were acquired using a Zeiss Observer Z.1 microscope in a 63x objectives. The images were processed with a deconvolution module using Zen software.

### Determination of mediators

The concentrations of cytokines in the supernatant obtained from cultures of infected B-1CDP cells or peritoneal macrophages were quantified after 24 hours of incubation by the method of sandwich immunoassay (ELISA) according to methodology recommended by the manufacturer (R&D). The optical density was obtained by reading in a plate spectrophotometer (Versamax Microplates Reader Molecular Devices, USA), with filter of 405 nm. The concentrations of cytokines were calculated from a standard curve of recombinant cytokines. Quantification of PGE_2_ was obtained by a PGE_2_ specific EIA kit, according to methodology recommended by the manufacturer (Cayman Chemical, Ann Arbor, MZ).

### Lipid bodies

To observe LBs, B-1CDP cells and macrophages were first fixed in 3,5% formalin in PBS for 30 min at room temperature, then stained with Nile red (Sigma) for 10 minutes (RT) at a final concentration of 10 mg/ml [35]. Following brief washes with PBS, and the slides were, stained with DAPI (Sigma). The morphology of fixed cells was observed, and Nile Red LBs were counted by light microscopy with a 100X objective lens in 50 consecutively scanned leukocytes.

### Statistical analysis

Statistical analysis was performed in the program GraphPad InStat version 3.01 (San Diego, USA). Data were analyzed by the method of Student’*t* test. Differences with a *p* value 0.05 or lower were considered significant.

## Results

### B-1CDP cells are more susceptible to infection with *L*. *major* in comparison with peritoneal macrophages

Macrophages correspond to the main reservoir of Leishmania infection *in vivo*. We evaluated the susceptibility/resistance to *L*. *major* infection of B-1CDP cells in comparison with peritoneal macrophages.

**Fig 1:**
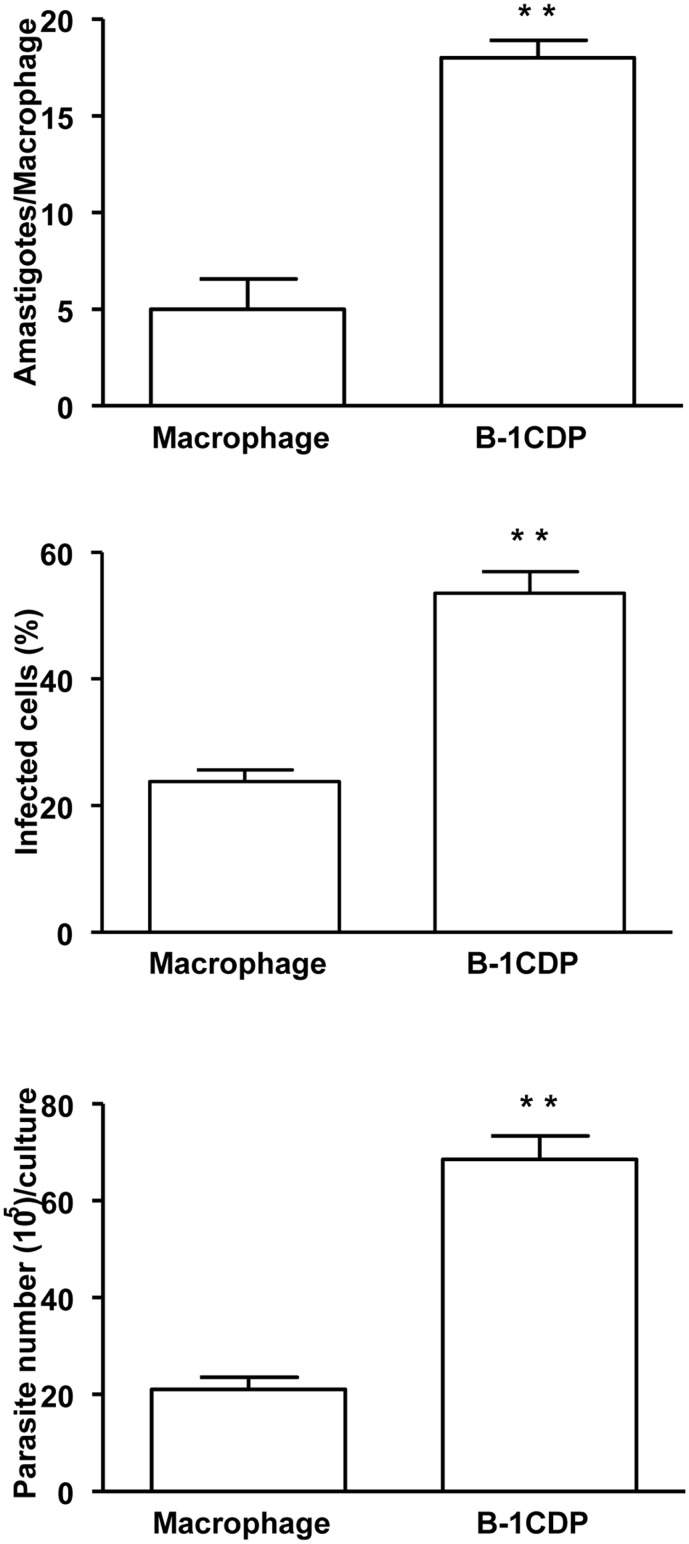
B-1CDP cells exhibit a susceptible phenotype to infection with *L*. *major in vitro* when compared to peritoneal macrophages. B-1CDP cells and peritoneal macrophages were cultured (10^5^/ml) and infected with metacyclic promastigotes of *L*. *major*. After 24 hours, the cell culture was washed and phagocytes were cultured for another 3 days with DMEM supplemented with 10% FBS at 37°C. After this period, cells were stained and amastigotes inside the phagocytes were counted under the light microscope (A) and set the percentage of infected cells (B). To quantify promastigotes forms in the supernatants, the cells were infected with *L*. *major*. After 24 hours of infection, cells were washed and cultured in Schneider medium for 5 days at a temperature of 27°C. After this period, the promastigotes were quantified in the supernatant of the cultures of infected phagocytes (C). All cultures were performed in triplicate and bars show the mean + SD. Statistical analysis were performed by T-Test from representative results of three similar experiments. **p ≤ 0.05.

B-1CDP cells and peritoneal macrophages were infected with *L*. *major* promastigotes and amastigotes were counted. Our results demonstrated that B-1CDP cells exhibit a greater number of intracellular amastigotes when compared with peritoneal macrophages (Fig 1A and S1 Fig) and were more permissive to infection, since they showed a higher percentage of infected cells (Fig 1B).

Furthermore, after 5 days of culture we observed a greater release of motile promastigotes forms of *L*. *major* in the supernatant of infected B-1CDP cells when compared to promastigotes present in the supernatant of peritoneal macrophages (Fig 1C). Despite of these differences, the parasite burden at 4 hours post-infection was identical for both cell types (S2 Fig).

### Detection of IL-10 in culture supernatants from infected B-1CDP cells controls *L*. *major* growth

It is known that B-1 cells produce IL-10 and use this cytokine as an autocrine growth factor [36]. Also this cytokine is an important regulator of macrophages [37]. Since B-1CDP cells are potent sources of IL-10, we decided to investigate whether IL-10 could be involved in infectivity of B-1CDP cells to *L*. *major in vitro*.

**Fig 2:**
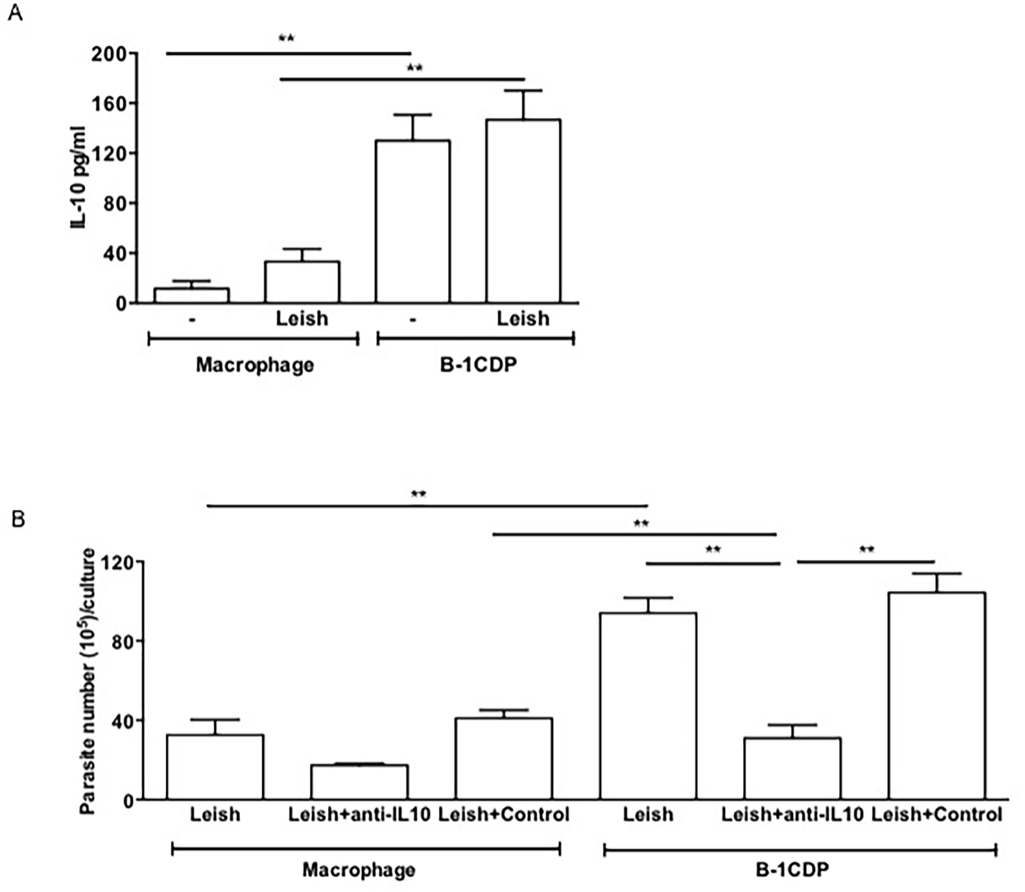
IL-10 is determinant for the increased parasite load in B-1CDP. B-1CDP cells and peritoneal macrophages were cultured in the presence or absence of *L*. *major* (MOI 10:1). After 24 hours, the supernatant was collected and IL-10 (A) was measured by ELISA. All cultures were performed in triplicate and bars show the mean ± SD. Representative result of three similar experiments **p <0.05. B-1CDP cells and peritoneal macrophages were treated or not with doses of monoclonal neutralizing anti-IL-10 or control isotype. Once were infected with *L*. *major*, after 24 hours, the cell cultures were washed with DMEM and incubated 3 days and then passed to Schneider medium. After 5 days in medium Schneider, promastigotes were counted in the supernatant (B). Statistical analysis were performed by Student’ *t* test from representative results of three similar experiments and bars show the mean +SD. **p ≤ 0.05.

The results shown in Fig 2A confirm that B-1CDP cells produce more IL-10 than peritoneal macrophages. We also evaluated the production of TGF-β, which is another important immunoregulatory factor produced by phagocytes [38–40]. However, our results did not show a remarkable production of TGF-β by B-1CDP cells (data not shown).

Next we evaluated the possible immunomodulatory role of IL-10 in the infection of B-1CDP cells in comparison with peritoneal macrophages. Our results showed that the use of a neutralizing anti-IL-10 antibody induced a remarkable reduction in parasitic load both in peritoneal macrophages and B-1CDP cells (Fig 2B). It is important to emphasize that the negative modulation induced by neutralization of IL-10 was more pronounced in cultures of infected B-1CDP cells. This result strongly suggests that there is indeed a close relationship between the susceptibility of B-1CDP cells to infection by *L*. *major* and the increased production of the cytokine IL-10.

### B-1CDP cells produce high amounts of lipid bodies

Lipid bodies are organelles consisting of a core of rich neutral lipids and wrapped with a monolayer of phospholipids, cholesterol, lipids, which contain a variety of proteins associated with different functions in cell metabolism, signaling, and inflammation [41]. Several groups have reported that host cells have their lipid metabolism altered and increase their number of lipid bodies following infection with intracellular pathogens [42–46]. Furthermore, our group has shown an increase of lipid bodies in macrophages infected with *Trypanosoma cruzi*, and their role in disease development [40].

**Fig 3:**
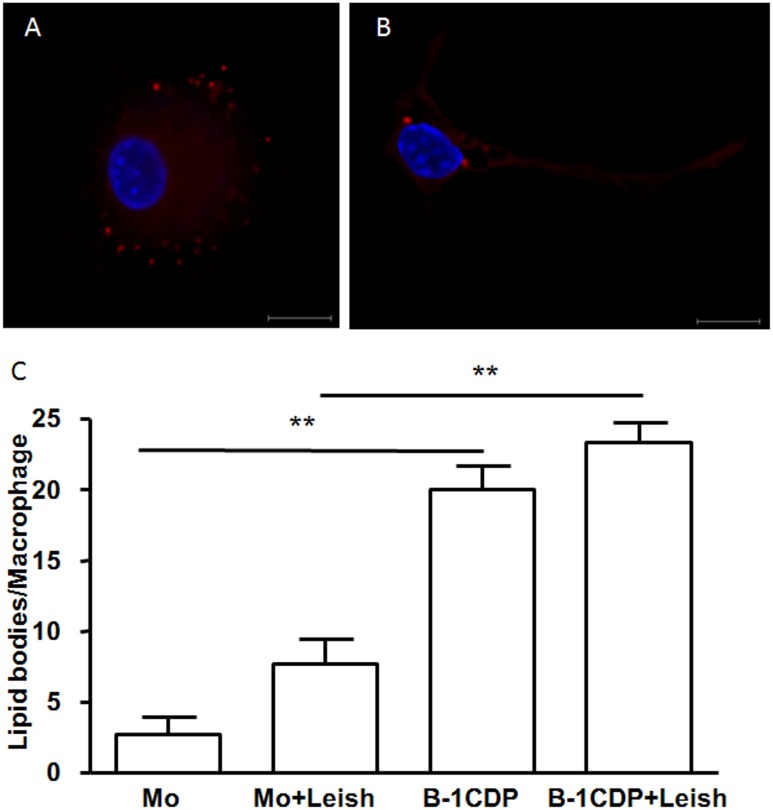
B-1CDP cells naturally express increased numbers of lipid bodies as compared to peritoneal macrophages. B-1CDP cells (A) and peritoneal macrophages (B) were incubated with glass coverslips, some cultures were infected with *L*. *major* (3C). Stained with Nile red (Sigma), the slides were washed and stained with DAPI (Sigma). The morphology of fixed cells was observed, and Nile red LBs were counted by light microscopy with a 100× objective lens in 50 consecutively scanned leukocytes. Statistical analysis were performed by Student’ *t* test from representative results of three similar experiments and bars show the mean +SD. **p ≤ 0.05. Bar, 10 μm. Representative of two experiments with identical results.

Based on this information, we investigated the presence of cytoplasmic lipid body organelles in B-1CDP cells and compared their number to peritoneal macrophages. We also investigated the generation of these organelles when peritoneal macrophages or B-1CDP cells were infected with *L*. *major*. Our data demonstrate that B-1CDP cells express a large number of lipid bodies in the cytoplasm even in the absence of stimulus (Fig 3A). After 24 hours of infection, we found that B-1CDP cells had a larger amount of lipid bodies compared to peritoneal macrophages (Fig 3B). However, infection per se did not increase statistically the number of lipid bodies in B-1CDP cells (Fig 3C).

### B-1CDP cells secrete high levels of PGE_2_

**Fig 4:**
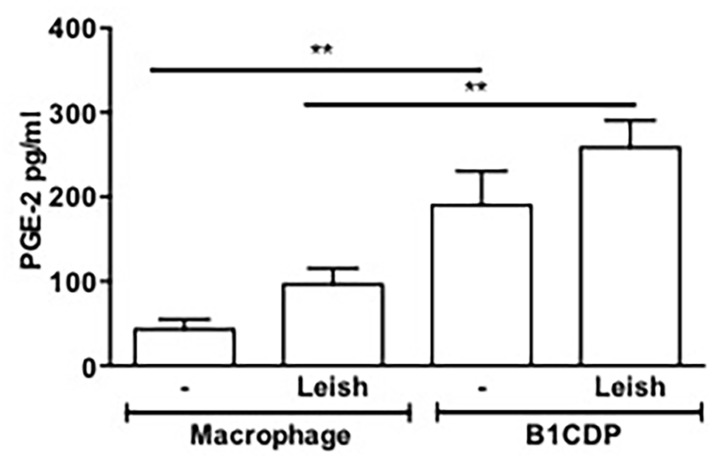
B-1CDP cells secrete high levels of PGE_2_ regardless the infection. B-1CDP cells and macrophages were incubated in the presence or absence of *L*. *major* (MOI 10:1). After 24 hours of infection the supernatant was collected, and PGE_**2**_ was measured by EIA. All cultures were performed in triplicate and bars show the mean ± SD. Statistical analysis were performed by Student’ *t* test from representative results of three similar experiments and bars show the mean +SD. **p ≤ 0.05.

Lipid bodies are cellular sites devoted to the synthesis of PGE_2_ [40]. Previous studies reported that Leishmania infection induces PGE_2_ production and that this production could favor the persistence and/or progression of disease [47]. Furthermore, B-1CDP cells constitutively express COX-1 and COX-2 enzymes, and produce considerable quantities of PGE_2_ in response to inflammatory signals [8]. Based on these data, we evaluated the involvement of lipid mediator PGE_2_ in infection of B-1CDP cells with *L*. *major*. For this purpose, we measured this lipid mediator in the culture supernatant after 24 hours of infection. As demonstrated in Fig 4, B-1CDP cells produce considerable amounts of PGE_2_ even in the absence of stimulus, and infection did not changing it significantly.

**Fig 5:**
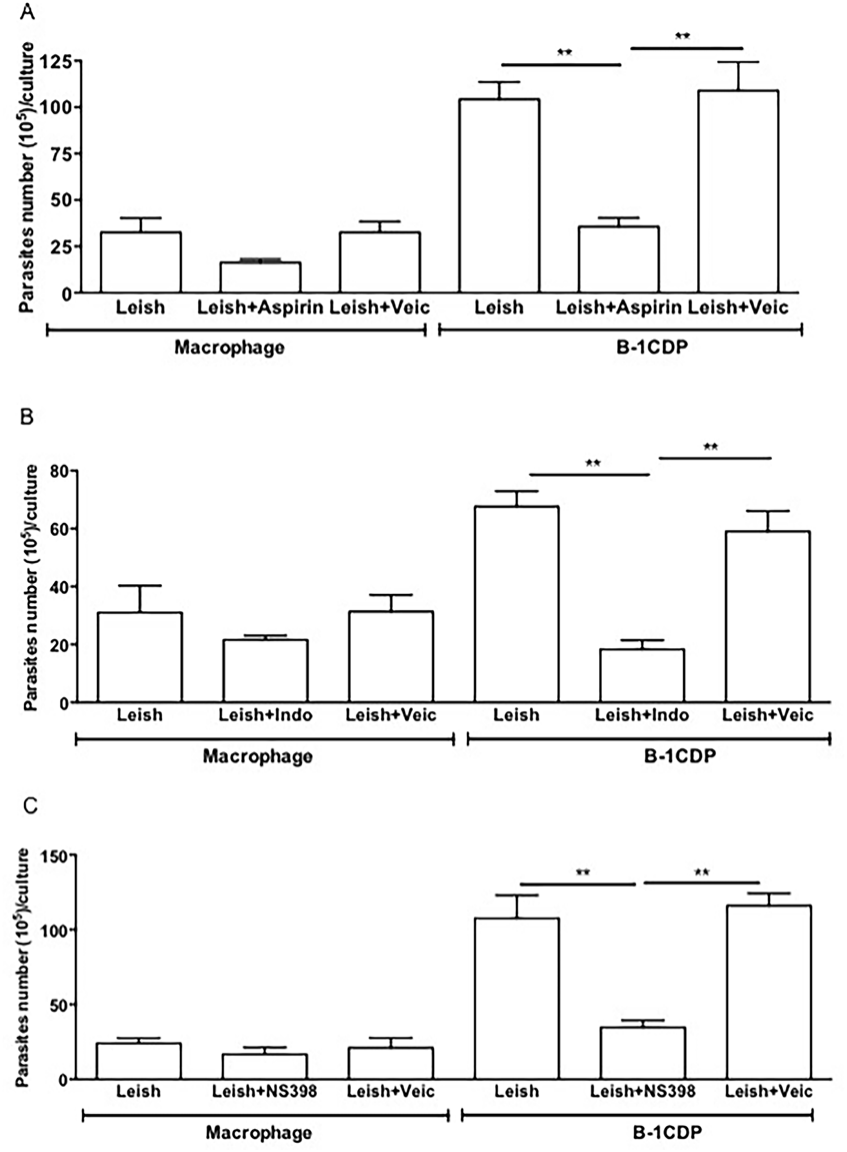
Blockage of the cyclooxygenase pathway shift the B-1CDP cells to protective phenotype in the *L major* infection. Macrophages and B-1CDP were incubated in the presence or absence of *L*. *major* treated or not with (A) aspirin (10 mg/mL), (B) indomethacin (1 mg/mL) or (C) NS-398 (1 mM). After 24 hours of incubation the cells were washed and incubated again for 5 days. After this time, the promastigotes were counted in the culture supernatant. All cultures were performed in triplicate and bars show the mean ± SD. Statistical analysis were performed by Student’ *t* test from representative results of three similar experiments and bars show the mean +SD. **p ≤ 0.05.

In order to determine whether PGE_2_ synthesis regulates parasite load, we used three different non-steroidal anti-inflammatory drugs (NSAIDs): Aspirin (Fig 5A), indomethacin (Fig 5B) and NS-398 (Fig 5C). The three drugs act as inhibitors of PGE_2_ production [38,48]. Our results demonstrate that inhibition of PGE_2_ production was accompanied by diminished parasite burden. It is important to emphasize that the inhibition was more pronounced in cultures of B-1CDP phagocytes treated with inhibitors (Fig 5A–5C). Treatment with NSAIDs drugs were not toxic to phagocytes, as measured by LDH release (S3 Fig).

### Effect of aspirin on IL-10 production and parasite release

**Fig 6:**
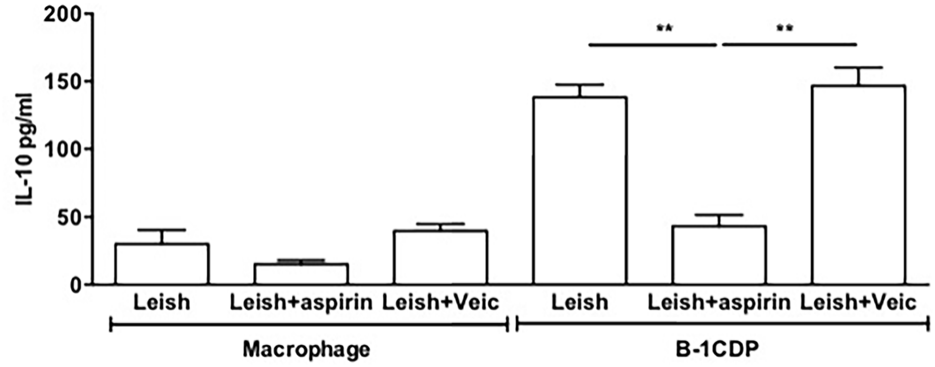
Effect of inhibition of PGE_2_ on IL-10 releases. Macrophages and B-1CDP cells were incubated with or without aspirin (10μg/mL) for 1 hour. They were then incubated in the presence or absence of *L*. *major* (MOI 10:1). After 24 hours of incubation, the supernatant was collected and IL-10 measured by ELISA. All cultures were performed in triplicate and bars show the mean ± SD. Statistical analysis were performed by Student’ *t* test from representative results of three similar experiments and bars show the mean +SD. **p ≤ 0.05.

After verifying the effect of cyclooxygenase inhibition on the parasite load, we investigated an effect on the production of IL-10 by B-1CDP. Our data demonstrate a significant decrease in the levels of IL-10 in the supernatant when we used NSAIDs in our system (Fig 6).

### B-1CDP cells from IL-10 KO mice are more competent to control *L*. *major* infection

**Fig 7:**
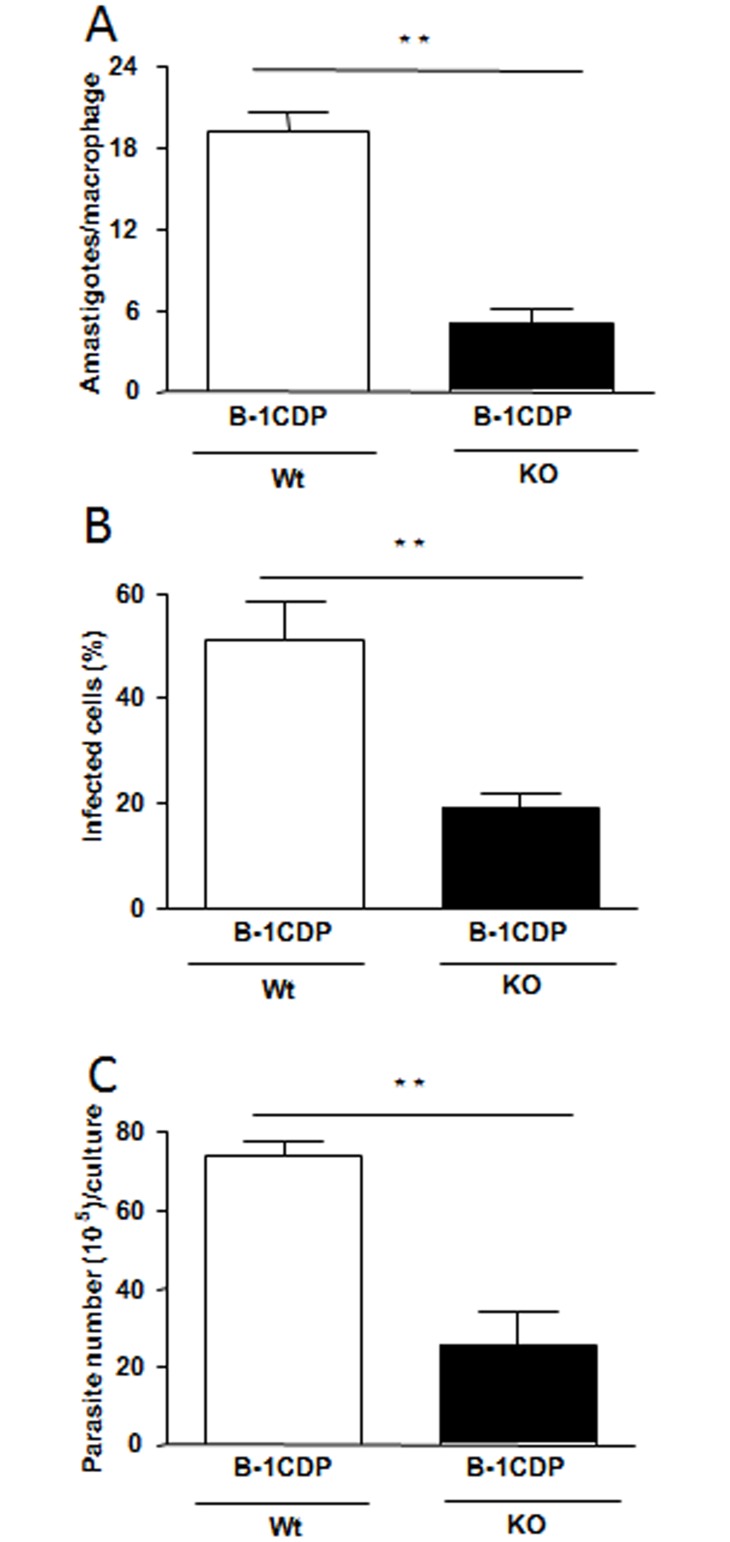
B-1CDP cells from IL-10 deficient mice are more competent to control *L*. *major* infection. To confirm the production of IL-10 is involved in the susceptibility to infection by *L*. *major*, we used B-1CDP cells from IL-10 KO mice. When compared to B-1CDP from wild type mice, the cells from IL-10 KO mice are more resistant to infection. Our data demonstrate decreased in the number of intracellular amastigotes (A) and percentage of infected cells (B). We also observed the significant decrease in the liberated promastigotes forms by B-1CDP from IL-10 KO mice (C). Statistical analysis were performed by Student’ *t* test from representative results of three similar experiments and bars show the mean +SD. **p ≤ 0.05.

To confirm that the production of IL-10 is involved in the susceptibility to infection by *L*. *major*, we used B-1CDP cells from IL-10 KO mice. When compared to B-1CDP from wild type mice, the cells from IL-10 KO mice were more resistant to infection. Our data demonstrated a decrease in the number of intracellular amastigotes (Fig 7A) and percentage of infected cells (Fig 7B). Also, we observed the significant decrease in the amount of promastigote forms released by B-1CDP cells from IL-10 KO mice (Fig 7C). However, we did not observe any differences in parasitic capture by B-1CDP cells 4 hours after infection (data not shown).

## Discussion

Phagocytosis is predominantly performed by professional phagocytes, such as polymorphonuclear cells, monocytes and macrophages [49]. Other types of cells such as fibroblasts and epithelial cells are also capable of phagocytizing particles, but in a much smaller degree compared to professional phagocytes.

Unlike professional phagocytes, most B cell subpopulations cannot perform phagocytosis [50]. However, several studies have discussed the mechanisms by which B-1 cells differentiate into phagocytes and the ontogenetic and physiological implications of this phenomenon. Reports in the literature have shown that pre-CD5+ B cells can differentiate into cells similar to macrophages [43,51].

Moreover, a link between the development of B-1 cells and macrophages was established by identifying bi-phenotypic B cell/macrophage progenitors in fetal liver [17] and adult bone marrow [51]. The presence of bi-phenotypic B cell/macrophage precursor in mammals suggests a close evolutionary relationship between B cells and macrophages derived from monocytes, which indicates the possibility of a common phylogenetic ancestor with attributes of both cell types, which has already been demonstrated in teleost fish [52].

Borrello and Phipps [6] demonstrated the differentiation of splenocytes simultaneously expressing B-cell and macrophage characteristics, when co-cultured with fibroblasts. Almeida and colleagues [33] showed that peritoneal B-1 cells proliferate and differentiate into new mononuclear phagocytic cells with no relation to blood derived monocytes. A further evidence for this B-1 cell/phagocyte link are discussed elsewhere [4].

In the present study, we demonstrate that phagocytes derived from B-1 cells (called B-1CDP) have the ability to phagocytose the pathogenic protozoan parasite *L*. *major*. Moreover, B-1CDP cells are more susceptible to infection with this parasite when compared with murine peritoneal macrophages; the intracellular load of amastigote can also be indirectly evaluated by the ability of host cells to release promastigotes following culture in Schneider medium [34]. The amount of released promastigotes in the supernatants after 5 days of infection was higher in cultures of the B-1CDP cells, as compared to cultures of murine peritoneal macrophages. We investigated which cytokines could be involved in this phenomenon. The cytokine IL-10 is produced by different cell types, such as macrophages, dendritic cells (DC), B cells and different subtypes of T lymphocytes [53,54]. Many of the effects of IL-10 on the functions of T cells and NK cells are now known to be mediated by a direct effect of IL -10 on macrophages. In addition, IL-10 may act directly on CD4+ T cells by inhibiting the production and proliferation of IL-2, IFN-γ, IL-4, IL-5 and TNF-α [53,55,56]. It is well characterized that IL-10 plays an important role in susceptibility to leishmaniasis. It suppresses the synthesis of the inflammatory cytokine IFN-γ and can inhibit the production of NO by activated macrophages and also down-regulates the expression of MHC class I and class II and co-stimulatory molecules (B7.1 and B7.2) on the surface of macrophages [57]. Furthermore, BALB/c mice deficient in IL-10 could control the *L*. *major* infection, suggesting that this cytokine might play an important role in mediating susceptibility to and pathogenesis of experimental cutaneous leishmaniasis [58].

The TGF-β is an important cytokine with anti-inflammatory activity, which was characterized as an important factor related susceptibility to experimental infection by Leishmania *in vivo* [59]. Our results indicated that the susceptibility to *L*. *major* infection presented by B-1CDP cells is related to the production of IL-10. Surprisingly, our results also indicated that the cytokine TGF-β is not involved in the increased infection of B-1CDP cells (data not shown).

B-1CDP cells produce a large quantity of IL-10 even without stimulation, in agreement with a marked anti-inflammatory profile [13,21], and with a propensity for infection by Leishmania. The role of IL-10 was confirmed by showing that a neutralizing antibody specific for IL-10 induced significant reduction in the amount of promastigotes released in culture supernatants.

Another aspect that could lead to increased susceptibility of B-1CDP cells to *L*. *major* infection, were the lipid bodies presented in that cell population. Lipid bodies are key organelles involved in cholesterol metabolism and the synthesis of fatty acids indicating that both anabolic and catabolic phases of lipid metabolism occur in these organelles [60,61]. Leukocytes have few lipid bodies, but these cells can be stimulated to form new lipid bodies quickly [62]. Accumulation of lipid bodies was also identified within peritoneal macrophages during the acute phase of experimental infection with the parasite *T*. *cruzi* and *L*. *amazonensis* [40,63]. Consistent with the role of inflammation on the leucocytes, the lipid bodies formed by these cells are important sites for the production of inflammatory mediators. Lipid bodies contain arachidonic acid, which serves as a precursor for the synthesis of eicosanoids, and all the enzymes necessary for this synthesis, including cyclooxygenase (COX), PGE_2_ synthase, 5/15-lipoxygenase (5-LO and 15-LO), and leukotriene C4 synthase [64].

As demonstrated in our study, the B-1CDP cells contain a large amount of cytoplasmatic lipid bodies compared with murine peritoneal macrophages. As the increased numbers of cytoplasmatic lipid bodies is associated to increase PGE_2_ production, the result on the *L*. *major* infection was expected. Our data shows that the inhibition of the PGE_2_ modulated the infection causing the decreased release of promastigotes forms by B-1CDP cells. PGE_2_ is known to be an important inducer of cytokines as TGF-β [38,48] and IL-10 [65].

The formation of lipid bodies within inflammatory macrophages positively correlates with increased generation of PGE_2_, indicating a role for these organelles in enhanced production of eicosanoids observed in Chagas´ disease [66]. The increased capacity of macrophages to generate PGE_2_ in the course of a pathogenic infection due to the increased formation of lipid bodies may contribute to mechanism by which intracellular pathogens survive in the host cells. The high concentration of PGE_2_ is a potent inhibitor of the T cell response and NO production by macrophages, favoring the growth of the intracellular parasite [38,39,67]. In addition, PGE_2_ is essential for the increased growth of parasites in macrophages that have ingested of apoptotic cells [38,48].

IL-10 produced by B-1CDP cells is the key to understanding their increased susceptibility to infection. Using B-1CDP cells from IL-10 KO mice the infection with *L*. *major* was less effective. These results strongly suggest that IL-10 secreted by B-1CDP cells, and probably B-1 cells as well, are involved in the susceptibility to infection. Popi and colleagues [68], using the *Coxiella burnetti* cell infection model *in vitro*, obtained a similar result. They found that peritoneal macrophages from BALB/c XID mice, which are deficient in B-1 cells, mice are more resistant to intracellular infection by *C*. *burnetti* when compared with peritoneal macrophages from wild-type BALB/c mice. The involvement of B-1 cells in experimental infection with Leishmania remains unclear, only two independent studies have reported the involvement of these cells in the infection, however the results were not conclusive [69,70].

Altogether, our data unveil that PGE_2_-driven production of IL-10 determines susceptibility of B-1CDP cells to *L*. *major* infection. Little is known about the physiology of B-1CDP cells, and their immune functions. The implications of our present findings in the pathophysiology of immunity remain open for further investigation. The “promiscuous" expression of both myeloid and lymphoid characteristics of a single cell type, and the factors governing differentiation of B-1 cells in B-1CDP cells will certainly open new avenues for the understanding of lymphoid and myeloid cells and their role in parasitic diseases.

## Supporting Information

S1 Fig B-1CDP cells exhibit a significant number of intracellular amastigotes compared with macrophages peritoneal. B-1CDP cells (A) and peritoneal macrophages (B) were cultured (10^5^/ml) and infected with promastigotes of *L*. *major*. After 24 hours, the cell culture was washed and phagocytes were cultured for another 3 days with DMEM supplemented with 10% FBS at 37°C. After this period, cells were fixed and permeabilized. Following incubation with Polyclonal goat antibody anti-mouse IgM-FITC (B-1CDP cells) and rat anti-mouse F4/80-FITC (macrophages). After incubation, glass cover slips were washed again and incubated in 1 microgram/mL of DAPI. After 10 min of incubation, glass cover slips were washed and mounted. Images were acquired using a Zeiss Observer Z.1 microscope in a 63x objectives. After the acquisition, images were processed with a deconvolution module using Zen software. Bar, 10 μm. Representative of two experiments with identical results.
(TIF)

S2 Fig Comparison of the susceptibility to *L*. *major* infection between B-1CDP cells and peritoneal macrophage. B-1CDP cells and peritoneal macrophages were cultured (10^5^/ml) and infected with metacyclic promastigotes of *L*. *major*. After 4 hours, the cell culture was washed and cells were stained and parasites inside the phagocytes were counted under the light microscope. All cultures were performed in triplicate and bars show the mean +SD.
(TIF)

S3 Fig Lactate dehydrogenase assay of infected B-1CDP cells and peritoneal macrophages in the presence of NSAIDs. B-1CDP cells and peritoneal macrophages were cultured (10^5^/ml) and infected with promastigotes of *L*. *major* were treated or not with aspirin (10 mg/mL), indomethacin (1 mg/mL) or NS-398 (1 mM). After 72 hours of incubation the supernatant was withdrawn and frozen for determination of LDH release.
(TIF)
