# Supplementary figures and images for: The PGE2/IL-10 Axis Determines Susceptibility of B-1 Cell-Derived Phagocytes (B-1CDP) to Leishmania major Infection
Source: PLoS One. 2015 May 1;10(5):e0124888. doi: 10.1371/journal.pone.0124888 (PMC4416734; doi:10.1371/journal.pone.0124888)

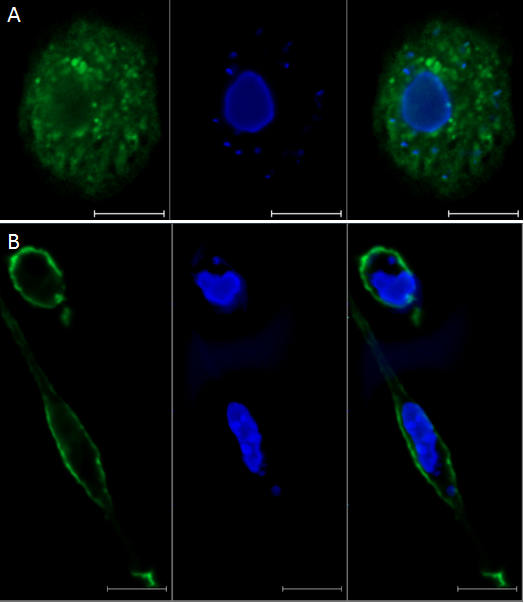

Supplement: S1 Fig — B-1CDP cells (A) and peritoneal macrophages (B) were cultured (105/ml) and infected with promastigotes of L. major. After 24 hours, the cell culture was washed and phagocytes were cultured for another 3 days with DMEM supplemented with 10% FBS at 37°C. After this period, cells were fixed and permeabilized. Following incubation with Polyclonal goat antibody anti-mouse IgM-FITC (B-1CDP cells) and rat anti-mouse F4/80-FITC (macrophages). After incubation, glass cover slips were washed again and incubated in 1 microgram/mL of DAPI. After 10 min of incubation, glass cover slips were washed and mounted. Images were acquired using a Zeiss Observer Z.1 microscope in a 63x objectives. After the acquisition, images were processed with a deconvolution module using Zen software. Bar, 10 μm. Representative of two experiments with identical results. (TIF) [file pone.0124888.s001.tif]

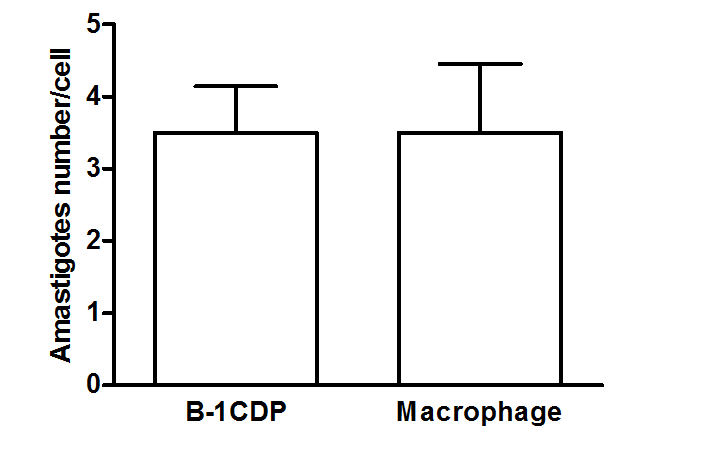

Supplement: S2 Fig — B-1CDP cells and peritoneal macrophages were cultured (105/ml) and infected with metacyclic promastigotes of L. major. After 4 hours, the cell culture was washed and cells were stained and parasites inside the phagocytes were counted under the light microscope. All cultures were performed in triplicate and bars show the mean +SD. (TIF) [file pone.0124888.s002.tif]

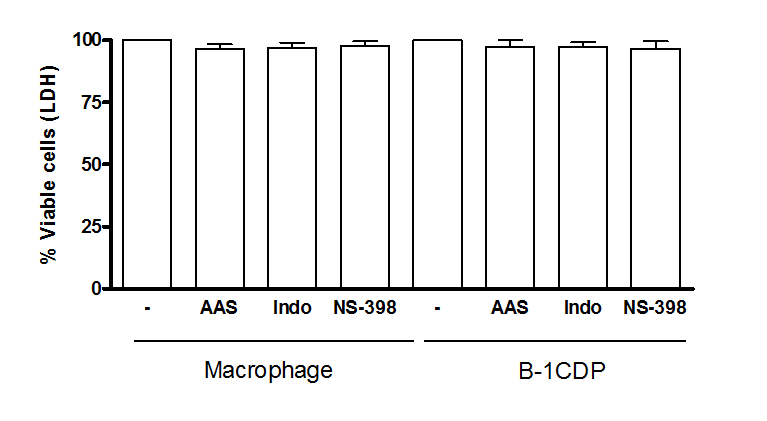

Supplement: S3 Fig — B-1CDP cells and peritoneal macrophages were cultured (105/ml) and infected with promastigotes of L. major were treated or not with aspirin (10 mg/mL), indomethacin (1 mg/mL) or NS-398 (1 mM). After 72 hours of incubation the supernatant was withdrawn and frozen for determination of LDH release. (TIF) [file pone.0124888.s003.tif]
